# Testing Genetic Association by Regressing Genotype over Multiple Phenotypes

**DOI:** 10.1371/journal.pone.0106918

**Published:** 2014-09-15

**Authors:** Kai Wang

**Affiliations:** Department of Biostatistics, University of Iowa, Iowa City, Iowa, United States of America; Indiana University Bloomington, United States of America

## Abstract

Complex disorders are typically characterized by multiple phenotypes. Analyzing these phenotypes jointly is expected to be more powerful than dealing with one of them at a time. A recent approach (O'Reilly et al. 2012) is to regress the genotype at a SNP marker on multiple phenotypes and apply the proportional odds model. In the current research, we introduce an explicit expression for the score test statistic and its non-centrality parameter that determines its power. Same simulation studies as those reported in Galesloot et al. (2014) were conducted to assess its performance. We demonstrate by theoretical arguments and simulation studies that, despite its potential usefulness for multiple phenotypes, the proportional odds model method can be less powerful than regular methods for univariate traits. We also introduce an implementation of the proposed score statistic in an R package named iGasso.

## Introduction

Complex human disorders are often characterized by multiple phenotypes. Some of them might be categorical while others might be continuous. For instance, patients with Bardet-Biedl syndrome often suffer from vision loss, hypertension and high cholesterol level caused by obesity, polydactyly, and other abnormalities. In order to map the genetic variants underlying such disorders, it is highly desirable to analyze all available phenotypes simultaneously. However, it is challenging to jointly model these phenotypes, especially when they are of different data types [1].

Let 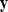 denote a 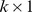 vector of 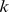 phenotypes on an individual and 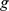 his/her genotype at a marker. If all the components of 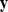 are continuous, one may use MANOVA given genetype 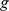. When the components of 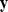 are of mixed data types, the choices are limited. One popular method is to first analyze each component individually and then combine the test statistics through a meta-analysis [2], [3]. These methods model the phenotype vector 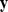 in terms of the genetic data 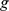.

For a single-nucleotide polymorphism (SNP), the distribution of its genotype 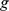 is trinomial. It is appealing to model 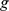 as a function of 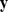 [4], [5]. Furthermore, since there is a natural ordering in the three genotypes at the SNP (assuming that the possibility of over-dominance is ignorable), one can use the ordinal logistic regression (a.k.a., the proportional odds model or the cumulative logit model) [6]. One immediate advantage of using the proportional odds model is that, unlike other methods, there is no need to make assumptions on the genetic effect such as additive, dominant, or recessive. The usefulness of this approach has been demonstrated via analyses of various data [5]. It is one of the best currently available multivariate methods [7]. However, it is the slowest one [7].

The test used in [5] is the likelihood ratio test (LRT). It involves numerical maximization under both the null hypothesis and the alternative hypothesis. We introduce a score test statistic using standard statistics theory. This statistic is asymptotically equivalent to the likelihood ratio test but computationally much faster due to the availability of its explicit expression, a feature useful in genome-wide association analysis. This explicit expression also gives insight on how the proportional odds model works in the context of genetic association analysis.

This report is organized as follows. We first introduce an explicit form of the score statistic and its non-centrality parameter. The form of this score statistic provides some insights on the ability of this method to detect association. The performance of the this score statistic is evaluated using the same simulation scenarios used in [7]. Finally, we consider an important case where the phenotype vector 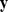 is univariate and binary to see how this test works for univariate phenotypes.

## Results

### The score statistic

The genetic data are assumed to come from a biallelic marker such as a single-nucleotide polymorphism (SNP). Let 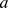 denote the reference allele and 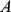 the other. For simplicity, we use 0, 1, and 2 to represent genotypes 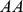, 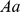, and 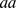, respectively. Regardless of the data types of the components of 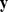, the genotype 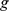 follows a trinomial distribution. In most cases, the effect of an allele is monotonic. That is, as the number of 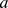 alleles increases from 0 to 2, the effect of genotypes 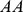, 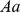, and 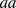 is non-decreasing or non-increasing. Over-dominance effect exists but is rather rare. Given this consideration, we model the genotype 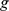 in terms of phenotype 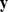 using the proportional odds model [5].

Let 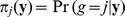 denote the probability that an individual's genotype 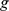 is 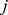 given phenotypic value 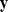. In the current situation, the proportional odds model models the cumulative probabilities 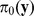 and 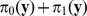 jointly as follows:
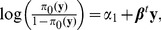
(1)

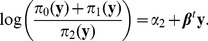
(2)


Here 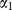 and 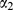 are intercepts and 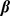 is a vector of coefficients. This model implies 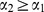 because 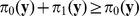. Since 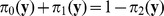, an alternative form of equation (2) is
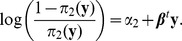



Equation (1) models the effect of phenotype y on the odds of genotype 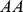 versus 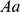 or 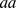 while equation (2) models the effect of phenotype y on the odds of genotype 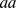 versus 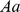 or 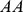. We have
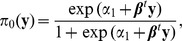


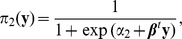
and 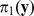 is determined by 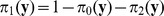. This model assumes that the difference of the left hand side of (1) or (2) for two phenotype vectors 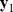 and 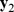 depends only on 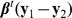 and is independent of genotype 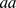 or 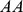:
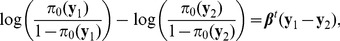
(3)

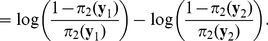
(4)


That is,
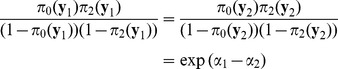
 does not depend on the value of 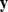.

Let 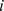 be the index for the 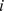
th individual in a sample of size 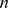, the log-likelihood function is
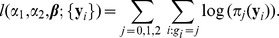



The hypotheses of interest are
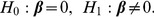
(5)


These hypotheses can be tested by the likelihood ratio statistic. To introduce the score statistic, define
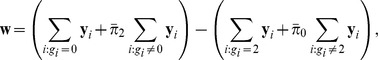
where 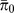 and 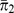 are the sample proportions of the genotypes for which 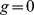 and 2, respectively. 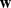 is the difference of two weighted summations of 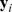. The summation in the first pair of parentheses weights 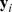 with 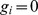 more than other 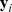
s (i.e., 1 versus 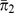) while the summation in the second pair of parentheses weights 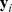 with 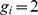 more (i.e., 1 versus 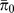). Let 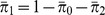. It is shown in the Methods section that a score statistic for testing hypotheses (5) is
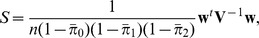
where
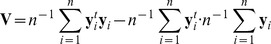
is the sample variance matrix of 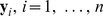. The non-centrality parameter of 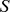 is
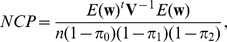
where the expectation in 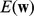 is taken under the alternative. This NCP can be used to compute power at significance level 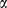 in the following way:
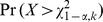
where 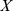 follows a chi-square distribution with 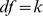 and non-centrality parameter 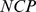 and 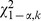 is the critical value from a chi-square distribution with 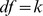 and non-centrality parameter 0.

### Simulation Study

The simulation study consists of two parts. In the first part, multivariate phenotypes were simulated the same way as in [7]. Genotype data at a single SNP were simulated with minor allele frequency 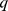 under the assumption Hardy-Weinberg equilibrium. Three phenotypes, denoted by 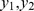, and 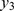, were simulated using the following relationship:
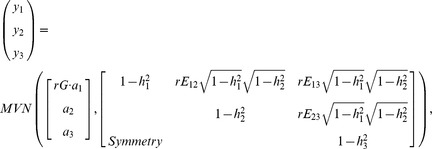



where 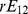, 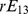, and 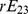 are pre-spedfied residual correlations between phenotypes excluding the QTL effect, 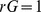 or 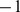 controls the effect direction of 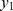 (those of 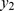 and 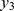 are fixed at 1) and 
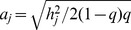
. Three scenarios were considered with only 1, 2, or all phenotypes were associated with the SNP. See [7] for more details. For each simulated data set, the LRT 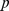-value is obtained from the R package MultiPhen and the score 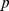-value is obtained from the R package iGasso. Empirical rejection rates over 1,000 replicates are reported in Table 1. The performance of the score test is very similar to that of LRT. The empirical power are very close to the power of LRT reported in [7].

**Table 1:** Non-centrality value and the associated power (presented in parentheses) for models used in the simulation studies.

	Frequency of Allele *a*	Effect of Allele *a*		
*K*	*p*	Recessive	Additive	Dominant
0.01	0.1	—	4.6780 (0.2597)	14.4110 (0.8387)
	0.3	2.8697 (0.1329)	9.7282 (0.6225)	18.5977 (0.9339)
	0.4	6.9507 (0.4323)	—	—
0.1	0.1	—	5.7000 (0.3374)	17.6383 (0.9182)
	0.3	3.4771 (0.1730)	11.7847 (0.7343)	22.5123 (0.9737)
	0.4	8.4233 (0.5379)	—	—

The relative genotype risks are 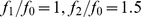 for recessive models; 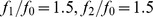 for dominant models; and 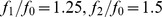 for additive models.

**Figure 1:**
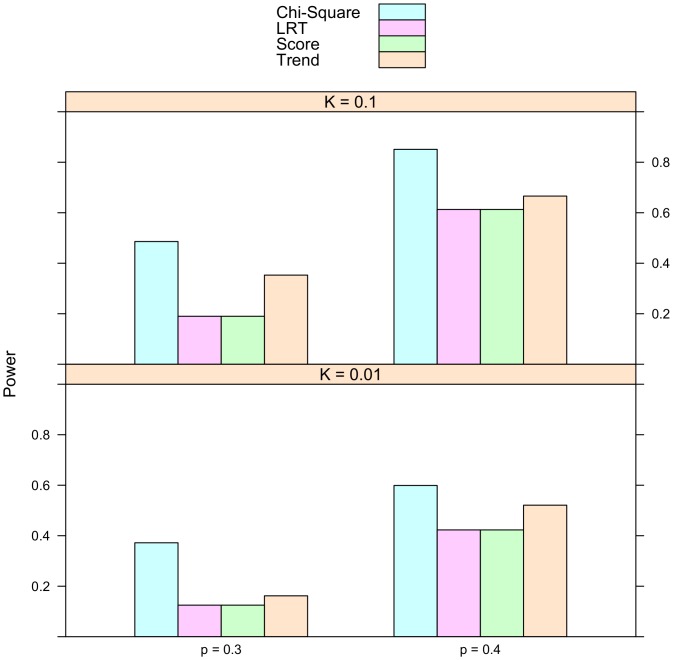
Simulated power for recessive model. The relative genotype risks are 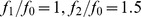. *K* represents disease prevalence and *p* is the frequency of allele *a*. The abbreviations for the test statistics are the same as in Table 2.

**Figure 2:**
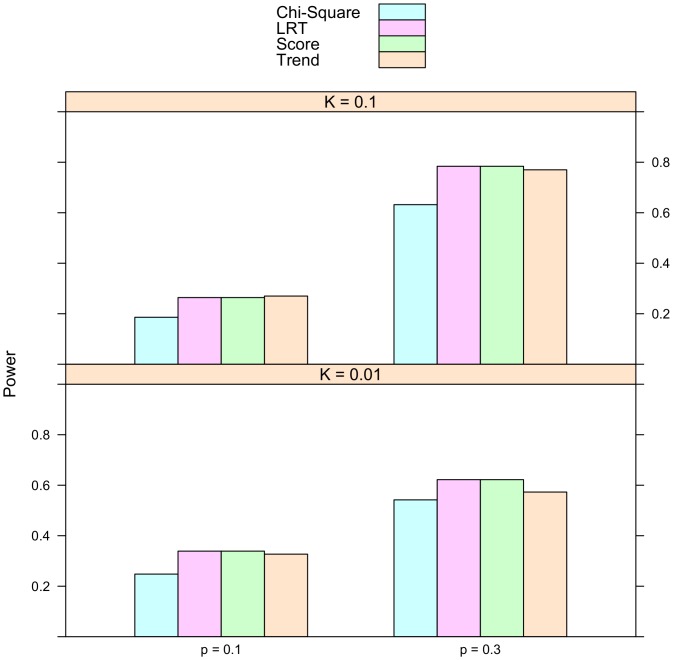
Simulated power for additive model. The relative genotype risks are 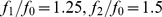. *K* represents disease prevalence and *p* is the frequency of allele *a*. The abbreviations for the test statistics are the same as in Table 2.

**Figure 3:**
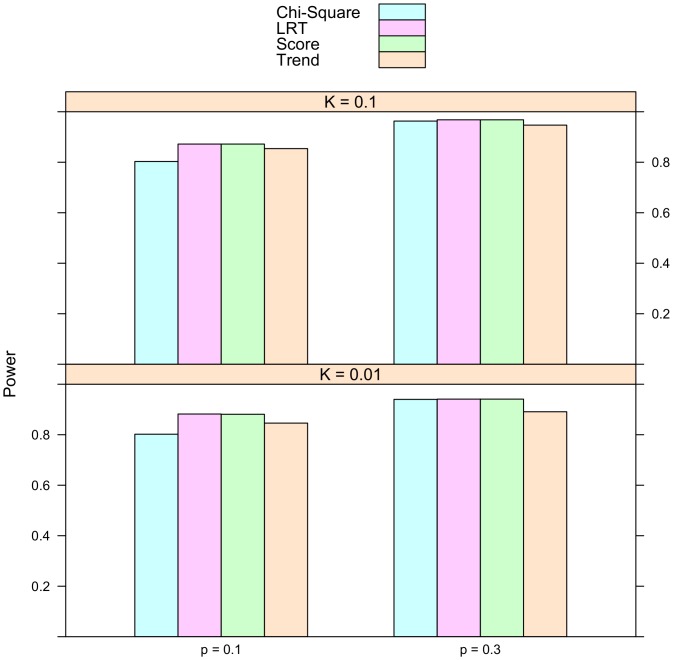
Simulated power for dominant model. The relative genotype risks are 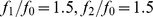. *K* represents disease prevalence and *p* is the frequency of allele *a*. The abbreviations for the test statistics are the same as in Table 2.

Is the proportional odds model always more powerful than the usual tests of association? To address this question, simulation were conducted on univariate phenotypes. The description of the simulation studies is provided in the Methods section. In addition to the proposed score statistic, the other test statistics used in the simulation include the Pearson's chi-square test, the Cochran-Armitage trend test, and the likelihood ratio test for the proportional odds model. The number of simulation replicates is fixed at 10,000. The sample size is 2,000. Half of the subjects are cases and half are controls. The simulated type I error rate for all these statistics is reported in Table 2. The empirical rejection rates are very close to their nominal levels, which are 0.1, 0.01, and 0.005. The simulated power is presented in Figures 1, 2, and 3. It is striking to see that for recessive models the proportional odds model is the least powerful. For instance, when prevalence 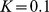 and minor allele frequency 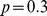, the power are 0.486 for Chi-Square test, 0.353 for Trend test, and 0.19 for both LRT and Score test. For other two models, there are situations it is more powerful than other methods. The simulated power for the 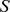 statistic is in line with the calculated power reported in Table 3.

**Table 2:** Simulated type I error rate in the case of a univariate phenotype under various generating models.

Penetrance (*K*)	Frequency of Allele *a* (*p*)	Significance Level	Test Statistic			
			Trend	Chi-Square	LRT	Score
0.01	0.1	0.1	0.085	0.095	0.091	0.091
		0.01	0.007	0.007	0.006	0.007
		0.005	0.005	0.004	0.004	0.004
	0.3	0.1	0.077	0.091	0.082	0.082
		0.01	0.009	0.008	0.010	0.010
		0.005	0.006	0.002	0.005	0.005
0.1	0.1	0.1	0.096	0.099	0.101	0.102
		0.01	0.011	0.009	0.010	0.010
		0.005	0.006	0.006	0.007	0.007
	0.3	0.1	0.116	0.117	0.109	0.108
		0.01	0.009	0.011	0.010	0.010
		0.005	0.006	0.007	0.003	0.003
0.01	0.1	0.1	0.085	0.095	0.091	0.091
		0.01	0.007	0.007	0.006	0.007
		0.005	0.005	0.004	0.004	0.004
	0.3	0.1	0.077	0.091	0.082	0.082
		0.01	0.009	0.008	0.010	0.010
		0.005	0.006	0.002	0.005	0.005
0.1	0.1	0.1	0.096	0.099	0.101	0.102
		0.01	0.011	0.009	0.010	0.010
		0.005	0.006	0.006	0.007	0.007
	0.3	0.1	0.116	0.117	0.109	0.108
		0.01	0.009	0.011	0.010	0.010
		0.005	0.006	0.007	0.003	0.003
0.01	0.1	0.1	0.085	0.095	0.091	0.091
		0.01	0.007	0.007	0.006	0.007
		0.005	0.005	0.004	0.004	0.004
	0.3	0.1	0.077	0.091	0.082	0.082
		0.01	0.009	0.008	0.010	0.010
		0.005	0.006	0.002	0.005	0.005
0.1	0.1	0.1	0.096	0.099	0.101	0.102
		0.01	0.011	0.009	0.010	0.010
		0.005	0.006	0.006	0.007	0.007
	0.3	0.1	0.116	0.117	0.109	0.108
		0.01	0.009	0.011	0.010	0.010
		0.005	0.006	0.007	0.003	0.003

The test statistics are: Trend — Cochran-Armitage trend test; Chi-Square — Pearson's chi-square test; LRT — the likelihood ratio test for the proportional odds model computed by using the polr function in the R package MASS; Score — the proposed score statistic computed by using the SNPass.test function in the R package iGasso.

**Table 3:** Simulated power under the same simulation scenarios of [7] over 1,000 replicates.

			Scenario					
			I		II		III	
*rG*	*rE*	MAF *q*	LRT	Score	LRT	Score	LRT	Score
1	0.0	0.01	0.125	0.125	0.184	0.183	0.278	0.278
		0.40	0.141	0.141	0.198	0.197	0.257	0.257
	0.3	0.01	0.127	0.129	0.185	0.185	0.184	0.183
		0.40	0.124	0.123	0.177	0.178	0.187	0.185
	0.7	0.01	0.221	0.221	0.268	0.267	0.129	0.129
		0.40	0.215	0.211	0.292	0.292	0.138	0.136
−1	0.0	0.01	—	—	0.202	0.201	0.289	0.288
		0.40	—	—	0.194	0.192	0.269	0.262
	0.3	0.01	—	—	0.267	0.266	0.344	0.344
		0.40	—	—	0.277	0.273	0.351	0.354
	0.7	0.01	—	—	0.589	0.589	0.694	0.694
		0.40	—	—	0.548	0.542	0.717	0.713

In scenario I, only phenotype 1 is associated with the SNP. *rG* = 1 or −1 does not affect the simulation results. Only results with *rG* = 1 are shown.

## Discussion

In this report, we introduced a score test statistic for the proportional odds model for testing the association between a SNP and multiple phenotypes and provided an implementation of this statistic. Same simulation studies as those reported in [7] were conducted to assess it performance. We also did simulation analyses to study the performance of proportional odds model for univariate phenotypes which is covered by [5]. Although appealing to studies on multiple phenotypes, this method may be less powerful for univariate traits than regular methods. For case-control data, our results suggest that the traditional Pearson's chi-square test and the Cochran-Armitage trend test are preferred when the disease allele frequency is less than 0.5 and the disease is recessive.

Nonetheless, the proportional odds model method provides a convenient way for analyzing multiple phenotypes, especially when these phenotypes are of different types [5]. If the proportional odds assumption is of concern, one may remove this assumption and adopt a multinomial logistic regression. For our simulation studies, the multinomial logistic regression would be equivalent to the Pearson's chi-square test statistic. There are quite few implementations of the multinomial logistic regression, for instance, the multinom function in R package nnet.

## Methods

### Derivation of the score statistic

The first-order derivatives of the log-likelihood function 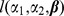 are
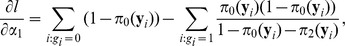


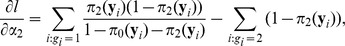


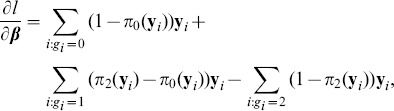
and the second-order derivatives are



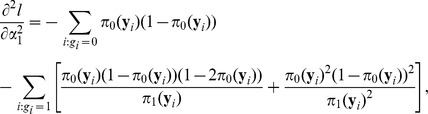





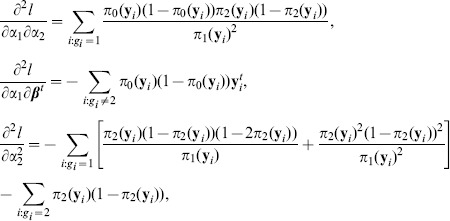


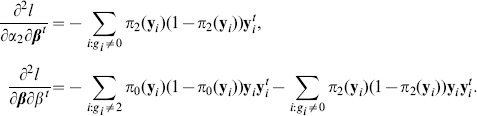



Under 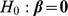, 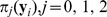 no longer depends on 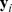. So their values are simply denoted by 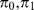, and 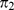, respectively. Let 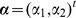. The expected Fisher information matrix evaluated at 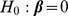 is
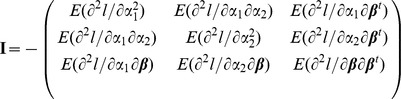


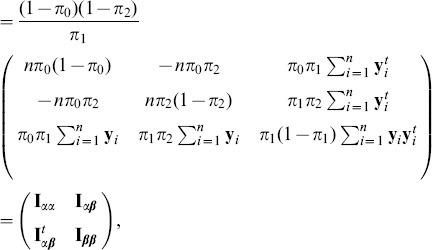



where the matrix partition is in an obvious manner. By standard asymptotic theory, the score statistic is
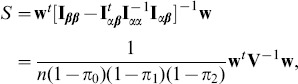



where 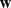 is 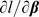 evaluated at 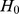:
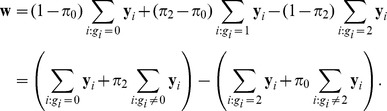



The unknown values of 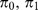, and 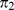 are estimated by their sample genotype proportions, respectively.

### Simulation Studies

Here is a description of the simulation procedure for the case of a dichotomous phenotype. Suppose the trait is Mendelian. Let 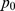, 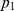, and 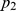 denote the frequencies of genotypes 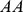, 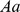 and 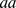 in general population and 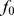, 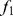, and 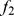 their penetrances, respectively. The prevalence of the disease would be 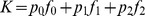. The genotype frequencies in cases are 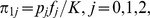 and in controls are 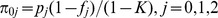. In this situation, the variance of 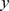 is 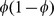 where 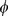 is the proportion of cases. The non-centrality parameter (NCP) of test statistic 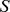 is equal to
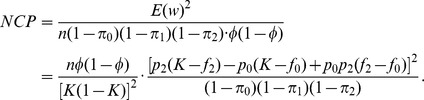



Let 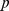 be the population frequency of allele *a*. Assuming Hardy-Weinberg equilibrium in the population, the frequencies of genotypes 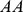, 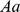, and 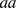 are 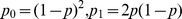, and 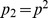, respectively. Let 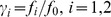, be the relative risk of genotype 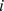 to genotype 0. A data generating model is completely determined by 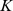, 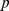, 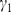, and 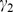. This is because 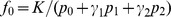, 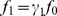, and 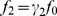. Hence the genotype frequencies in cases and controls are determined and data can be simulated. We consider a dominance model (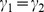), a recessive model (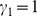), and an additive model (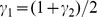). The NCPs for the models used in simulation are reported in Table 3. So are the power associated with these NCPs.

### R function SNPass.test

The R function SNPass.test in the package iGasso implements the proposed score statistic. R users can download and install iGasso from CRAN (http://cran.r-project.org/) or any CRAN mirror.

## References

[1] Zhu W, Zhang H (2009) Why do we test multiple traits in genetic association studies. Journal of the Korean Statistical Society 38: 1–10. 19655045 10.1016/j.jkss.2008.10.006 PMC2719985

[2] Xu X, Lu Tian L (2003) Combining dependent tests for linkage or association across multiple phenotypic traits. Biostatistics 4: 223–229. 12925518 10.1093/biostatistics/4.2.223

[3] Yang Q, Wu H, Guo CY, Fox CS (2010) Analyze multivariate phenotypes in genetic association studies by combining univariate association tests. Genet Epidemiol 34: 444–454. 20583287 10.1002/gepi.20497 PMC3090041

[4] Wang K, Huang J (2011) Treating phenotype as given: A simple resampling method for genome-wide association studies. Genetic Analysis Workshop 17 5: S60. 10.1186/1753-6561-5-S9-S60 PMC3287899 22373312

[5] O'Reilly PF, Hoggart CJ, Pomyen Y, Calboli FCF, Elliott P, et al (2012) MultiPhen: Joint model of multiple phenotypes can increase discovery in GWAS. PLoS ONE 7: e34861. 22567092 10.1371/journal.pone.0034861 PMC3342314

[6] Agresti A (2002) Categorical Data Analysis. John Wiley & Sons, Inc., 2nd edition.

[7] Galesloot TE, van Steen K, Kiemeney LALM, Janss LL, Vermeulen SH (2014) A comparison of multivariate genome-wide association methods. PLoS ONE 9: e95923. 24763738 10.1371/journal.pone.0095923 PMC3999149

